# Novel Structural Motif To Promote Mg-Ion Mobility: Investigating ABO_4_ Zircons as Magnesium Intercalation Cathodes

**DOI:** 10.1021/acsami.3c05964

**Published:** 2023-07-11

**Authors:** Ann Rutt, Dogancan Sari, Qian Chen, Jiyoon Kim, Gerbrand Ceder, Kristin A. Persson

**Affiliations:** † Department of Materials Science and Engineering, University of California, Berkeley 94720, United States; ‡ Materials Sciences Division, Lawrence Berkeley National Laboratory, Berkeley 94720, United States

**Keywords:** cathodes, magnesium batteries, energy storage, diffusion, multivalent ion mobility

## Abstract

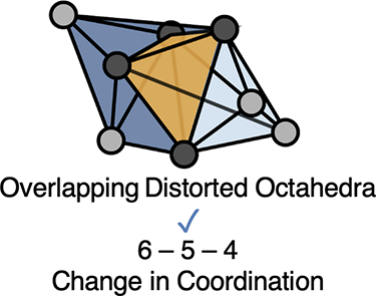

There is an increasing need for sustainable energy storage solutions as fossil fuels are replaced by renewable energy sources. Multivalent batteries, specifically Mg batteries, are one energy storage technology that researchers continue to develop with hopes to surpass the performance of Li-ion batteries. However, the limited energy density and transport properties of Mg cathodes remain critical challenges preventing the realization of high-performance multivalent batteries. In this work, ABO_4_ zircon materials (A = Y, Eu and B = V, Cr) are computationally and experimentally evaluated as Mg intercalation cathodes. Remarkably good Mg-ion transport properties were predicted and Mg-ion intercalation was experimentally verified in sol–gel synthesized zircon YVO_4_, EuVO_4_, and EuCrO_4_. Among them, EuVO_4_ exhibited the best electrochemical performance and demonstrated repeated reversible cycling. While we believe that the one-dimensional diffusion channels and redox-active species tetragonal coordination limit the value of many zircons as high-performance cathodes, their unique structural motif of overlapping polyhedra along the diffusion pathway appears instrumental for promoting good Mg-ion mobility. The motif results in a favorable “6-5-4” change in coordination that avoids unfavorable sites with lower coordination along the diffusion pathway and a structural design metric for future Mg cathode development.

## Introduction

Multivalent batteries are one of several emerging “beyond Li-ion” battery energy storage technologies that aim to enable large-scale renewable energy.^1−4^ Of the multivalent battery chemistries (Mg^2+^, Ca^2+^, Zn^2+^, Al^3+^, etc.), the most progress has been made with magnesium batteries since the first lab-scale prototype magnesium cell was reported in 2000 using a Mg_*x*_Mo_6_S_8_ Chevrel cathode.^1^ Research efforts continue to focus on improving Mg cathodes in order to identify materials with suitable energy density and rate capability for high-performance batteries.^6−8^ While progress has been made, the best available Mg cathodes exhibit inferior voltages as compared to state-of-the-art Li cathodes, and poor solid-state mobility, which results in insufficient rate capability.^2^ The identification of high-performance Mg cathodes is an issue that must be overcome in order to realize a Mg battery chemistry that can outperform Li-ion batteries and warrant commercialization.^3−5^

Given the transport challenges inherent to more polarizing multivalent ions (compared to Li^+^), recent efforts have been dedicated to understanding and improving the solid-state mobility of multivalent ions, especially in oxide hosts. Common material design strategies^5^ include: (1) using materials with larger anions, which allow for better screening, in the host framework (e.g., opting for sulfides or selenides over oxides) and (2) leveraging the coordination preference of a specific cation to improve transport. For example, Mg^2+^ has a strong preference for octahedral local bonding environments,^6,7^ which usually results in poor mobility and difficult extraction of Mg^2+^ from octahedral sites.

**Figure 1:**
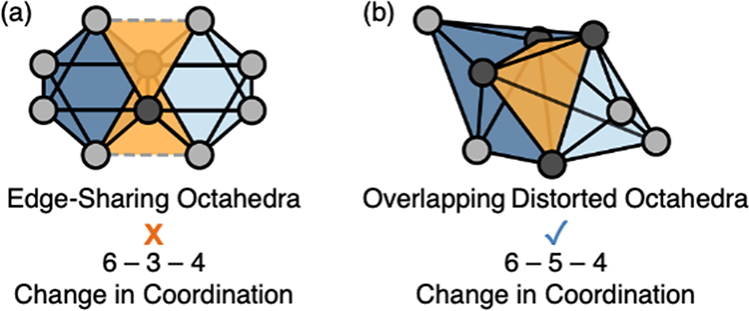
Visual representation of the characteristic structural motifs along diffusion pathways where the gray circles represent anions. Neighboring octahedral sites are colored with different shades of blue, while the intermediate tetrahedral sites are colored yellow. The darker gray-colored circles indicate which anions are shared by both octahedra. Panel (a) shows the edge-sharing octahedral motif found in previously studied cathodes (e.g., spinels, layered structures, and olivines) where there is no shared volume with the intermediate tetrahedral site. Panel (b) shows the overlapping distorted octahedral motif characteristic of zircons where volume is shared with the intermediate tetrahedral site.

High energy sites along the diffusion pathway in a material can also correspond to the mobile cation passing through points of lower coordination. These lower coordination points may represent a position where the mobile cation passes through a plane of neighboring anions.^2^ For example, in the structural motif where a diffusion pathway is composed of edge-sharing octahedra as illustrated in Figure 1a, the lowest coordination occurs when the mobile cation passes through a triangular plane of 3 anions. This triangular plane is the shared face between the octahedral and intermediate tetrahedral site and corresponds to the highest energy point along the migration pathway in several materials with this edge-sharing octahedral motif.^8^ Materials with larger anions of the same charge are better at screening unfavorable electrostatic interactions at these bottlenecks which usually results in improved transport properties.^9−11^ While understanding the connection between various material’s properties and multivalent ion transport is evolving, identifying materials with better transport properties based on these principles remains a challenge.

**Figure 2:**
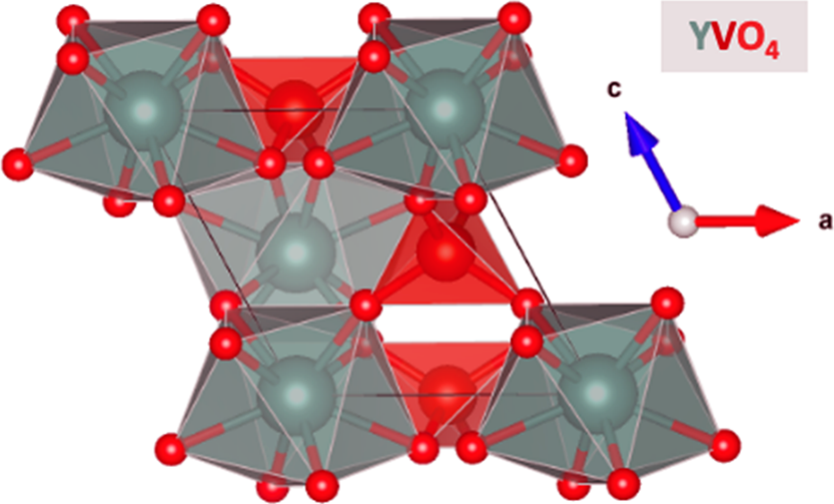
Unit cell structure of YVO_4_ showing the ABO_4_ zircon-type structure (with tetragonal space group *I*41/*amd*) composed of edge-sharing alternating AO_8_ dodecahedrons and BO_4_ tetrahedrons.

Our work introducing a computational screening approach to identify high-performance multivalent intercalation cathodes^9^ has proved instrumental in evaluating solid-state mobility in a wider variety of structure types. A new family of materials with the ABO_4_ zircon-type structure (with tetragonal space group *I*41/*amd*) was identified using this methodology, specifically for their predicted high Mg^2+^ mobility. Our subsequent investigation of these materials as Mg cathodes is reported in this work. The ABO_4_ zircon structure is composed of edge-sharing alternating AO_8_ dodecahedrons and BO_4_ tetrahedrons and is illustrated in Figure 2, a depiction of the unit cell structure of zircon YVO_4_. In the AO_8_ dodecahedron, the A atom is 8-coordinated, while in the BO_4_ tetrahedron, the B atom is 4-coordinated. These structures also exhibit interstitial sites of distorted octahedra and tetrahedrons, forming one-dimensional channels,^12^ which are presented in Figure 1b.

As illustrated in Figure 1, zircons present a unique structural motif compared to many previously studied cathodes (e.g., spinels, layered structures, and olivines) with edge-sharing octahedral sites.^8^ A key difference is that the tetrahedral and distorted octahedral interstitial sites of the zircon structure are overlapping in volume, in contrast to the absence of shared volume in materials with edge-sharing octahedral interstitial sites, connected by face-sharing tetrahedral sites. The prototype zircon is ZrSiO_4_, a naturally occurring mineral,^13^ although ABO_4_ zircons span a wide range of chemistries. This work focuses on a smaller subset of zircons (A = Y, Eu and B = V, Cr) which contain a redox-active cation and can be synthesized through previously reported methods.

The four zircon materials, which are the focus of this work, have been available in the Materials Project with standard properties calculated by density functional theory (DFT).^14^ In addition, the structural, mechanical, electronic, magnetic, and optical properties of zircons have previously been investigated, including YVO_4_,^15,16^ EuVO_4_,^17−19^ YCrO_4_,^13,20^ and EuCrO_4_.^21,22^ Comparatively, there is less work regarding the electrochemical and transport properties of zircons required to inform their performance as intercalation cathodes. Oxygen diffusivity has been measured for zircon EuVO4^18^ and the conductivity of interstitial H^+^, Li^+^, Na^+^, Mg^2+^, and Ca^2+^, was studied in zircon YPO_4_^23,24^ (although YPO_4_ lacks a redox active species which is one of the requirements for a cathode). To our knowledge, this is the first reported work to consider zircon materials as intercalation cathodes for Mg.

## Results

### Predicted Phase Stability upon Mg Intercalation

A previously developed computational method and associated workflow to predict insertion sites (here denoted the “insertion algorithm”)^25^ for host materials was used to evaluate the maximum viable concentration of Mg that could be introduced in zircon YVO_4_, EuVO_4_, YCrO_4_, and EuCrO_4_. This workflow performs successive DFT calculations of Mg-ion insertions at candidate interstitial sites, which are identified by charge density minima in the host structure determined by DFT calculations. The insertions are deemed successful as long as the relaxed discharged structure is similar (topotactic insertion) to the host structure as identified with the structure matcher module in pymatgen^26^ and does not exceed the redox capability based on the contained transition metal. Given these criteria, this capacity should be taken as an upper bound for the real capacity that can be achieved.

The “energy above hull” measures the driving force for a phase to decompose into potentially more stable phases. For a given material, it is measured as the energy per atom above the convex energy hull defined by the most stable phases in the relevant chemical space.^27,28^ The minimum value of this quantity, 0 eV/atom, indicates that a material is predicted to be the most thermodynamically stable phase at 0 K based on DFT calculations. Energy above hull values were calculated with the MP2020 compatibility scheme^29^ and the Materials Project^14^ database phase diagrams using pymatgen.^26^ The energy above hull values for the Mg_*x*_ABO_4_ zircons of interest (A = Y, Eu and B = V, Cr) are reported for 3 magnesium concentrations (*x* = 0, 0.5, 1) in Table 1. In addition, if a material is not in the most stable phase, the predicted decomposition products are included. Conversion voltages for the four zircons of interest were also calculated using pymatgen^26^ and phase diagrams from the Materials Project.^14^ The conversion voltages and their corresponding reactions are shown in Table 2.

**Table 1:** Energy Above Hull Values from DFT Calculations Combined with Materials Project^14^ Data to Evaluate Phase Stability for Mg_*x*_ABO_4_ Zircons (A = Y, Eu and B = V, Cr) at *x* = 0, 0.5, 1 Magnesium Concentrations^a^

ABO_4_ Zircon	Phase Stability (meV/atom)			Decomposition Products
	ABO_4_	Mg_0.5_ABO_4_	MgABO_4_	
YVO_4_ (mp-19133)	0	133	196	YVO_3_ (mp-18883)
				MgO (mp-1265)
EuVO_4_ (mp-22796)	0	0		
YCrO_4_ (mp-18825)	∼0^b^	139		Y_2_O_3_ (mp-2652)
				YCrO_4_ (mp-18825)
				MgCr_2_O_4_ (mp-19202)
				MgCrO_4_ (mp-19120)
EuCrO_4_ (mp-22586)	0.1	127	219	Eu_2_O_3_ (mp-1182469)
				EuCrO_4_ (mp-22586)
				MgCr_2_O_4_ (mp-19202)
				MgO (mp-1265)

a The decomposition products are included when a material was not in the most stable phase at that composition.

b Within our numerical accuracy, YCrO_4_ is degenerate with the hull.

**Table 2:** Conversion Reactions and Voltages Calculated from DFT for ABO_4_ Zircons (A = Y, Eu and B = V, Cr)

Conversion Reaction	Conversion Voltage (V)
2YVO_4_ + 2Mg → 2YVO_3_ + 2MgO	1.7
2EuVO_4_ + Mg → Eu_2_MgV_2_O_8_	1.9
2YcrO_4_ + 1.25Mg → Y_2_O_3_ + 0.5MgCrO_4_ + 0.75MgCr_2_O_4_	2.8
2EuCrO_4_ + 2Mg → MgCr_2_O_4_ + Eu_2_O_3_ + MgO	2.6

The insertion algorithm identified a single Mg-ion insertion per unit cell (2 formula units) in EuVO_4_ and YCrO_4_, resulting in a maximum intercalation level of Mg_0.5_ABO_4_. A maximum intercalation level of MgABO_4_ without any significant change in structure could be tolerated for YVO_4_ and EuCrO_4_, despite the high energy above the hull. The ABO_4_ ⇔ MgABO_4_ reaction is found to be more energetically favorable than ABO_4_ ⇔ Mg_0.5_ABO_4_ which makes observing Mg_0.5_ABO_4_ unlikely. With the exception of EuVO_4_, all energy above hull values upon magnesiation are >100 meV/atom, which strongly indicates that further magnesiation is unfavorable and will result in phase decomposition.^30,31^ The best phase stability was found in EuVO_4_, where both EuVO_4_ and Mg_0.5_EuVO_4_ were found to be the ground states with an energy above the hull of 0 meV/atom. The conversion voltages of YCrO_4_ (2.8 V) and EuCrO_4_ (2.6 V) are also significantly higher than those of YVO_4_ (1.7 V) and EuVO_4_ (1.9 V). Therefore, of the four zircons evaluated with DFT, Mg-ion intercalation is predicted to be most favorable in EuVO_4_.

### DFT-Predicted Battery Electrode Properties

**Figure 3:**
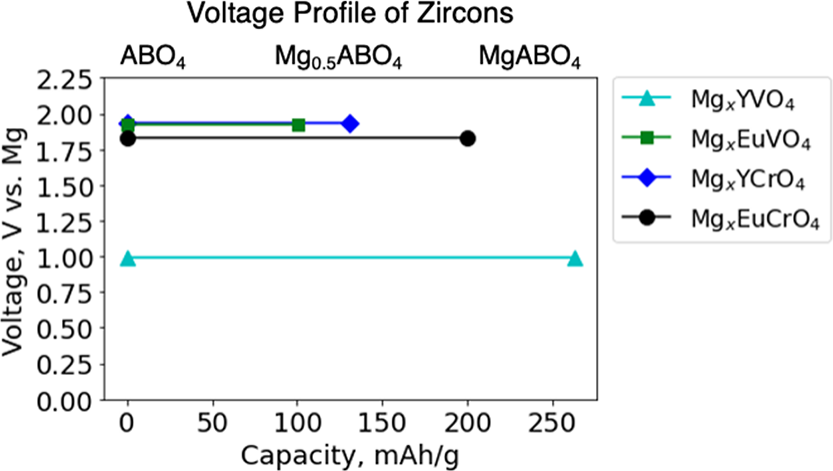
Theoretical voltage profiles based on DFT calculations for zircon YVO_4_, EuVO_4_, YCrO_4_, and EuCrO_4_ as Mg intercalation electrodes.

The Python package, pymatgen,^26^ was used to analyze the Mg electrode properties that can be determined from the DFT calculations generated by the insertion algorithm. These properties are reported in Table 3, which include voltage (compared to the Mg/Mg^2+^ redox couple), gravimetric capacity (based on the ABO_4_ molar mass for the charged composition without Mg), and the percent change in volume of the material’s crystal structure between the charged and discharged states. The voltage and gravimetric capacity values are plotted in Figure 3 to show the corresponding theoretical voltage profiles based on DFT calculations for zircon YVO_4_, EuVO_4_, YCrO_4_, and EuCrO_4_ as Mg intercalation electrodes. The gravimetric capacities of EuVO_4_ and YCrO_4_ are significantly lower than those of YVO_4_ and EuCrO_4_ due to their lower maximum intercalation level of Mg_0.5_ABO_4_. Similarly, the predicted volume changes of YVO_4_ and EuCrO_4_ are greater than EuVO_4_ and YCrO_4_ due to their higher maximum intercalation level of MgABO_4_.

**Table 3:** Summary of Theoretical ABO_4_ Zircon Electrode Properties Calculated Using DFT Such as Voltage (Compared to the Mg/Mg^2+^ Redox Couple), Gravimetric Capacity (Based on the ABO_4_ Molar Mass for the Charged Composition without Mg), and the Change in Volume of the Material’s Crystal Structure between the Charged and Discharged State

ABO_4_ Zircon	Intercalation Reaction	Voltage (V vs Mg/Mg^2+^)	Gravimetric Capacity (mAh/g)	Volume Change (%)
YVO_4_ (mp-19133)	Mg + YVO_4_ ⇔ MgYVO_4_	1.0	263	12
EuVO_4_ (mp-22796)	0.5 Mg + EuVO_4_ ⇔ Mg_0.5_EuVO_4_	1.9	100	7
YCrO_4_ (mp-18825)	0.5 Mg + YCrO_4_ ⇔ Mg_0.5_YCrO_4_	1.9	131	6
EuCrO_4_ (mp-22586)	Mg + EuCrO_4_ ⇔ MgEuCrO_4_	1.8	200	10

### DFT-Predicted Mg-Ion Mobility

**Figure 4:**
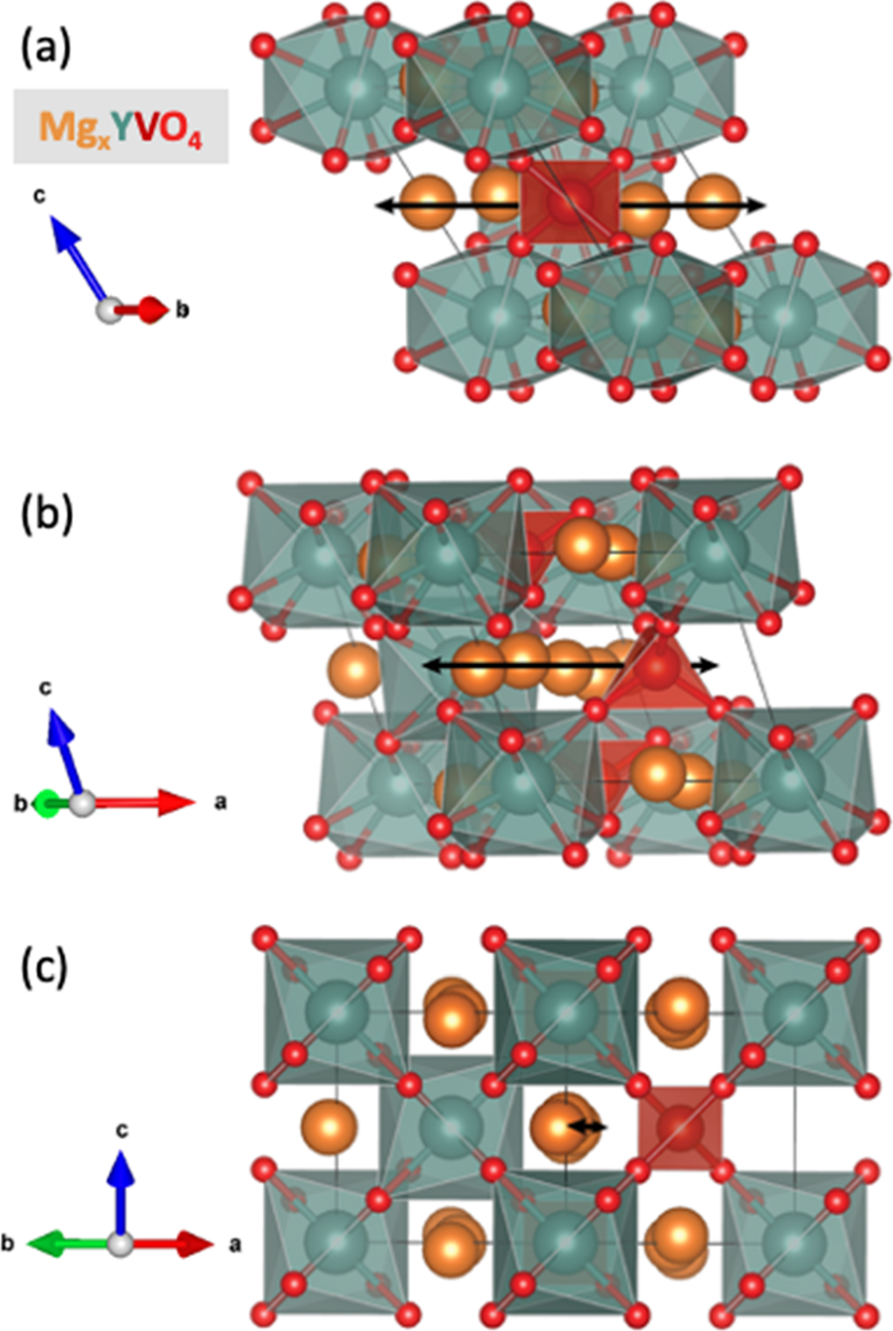
YVO_4_ zircon supercell illustrating the one-dimensional linear diffusion pathway shown from three different perspectives. In (a) the diffusion pathway is in the plane of the page, while in (c), the structure is rotated 90° and the diffusion pathway is perpendicular to the plane of the page. Panel (b) shows an intermediate perspective rotated 45° from (a) and (c).

The Mg sites identified by the insertion algorithm can be used to form a migration graph mapping out a network of connected sites in the host structure.^32^ This migration graph analysis enables searching for possible percolating pathways in the intercalation material. In this case, the insertion algorithm and migration graph analysis identified linear pathways consisting of the interstitial sites formed by distorted octahedra and tetrahedra. These sites form one-dimensional channels, which have been previously reported in a crystallography study of the zircon structure.^12^ Migration along this percolating pathway is expected to be composed of a series of repeating linear hops between interstitial sites that are approximately 1.5 Å apart. Notably, at the highest Mg concentration, we observed a vacant site between occupied Mg sites along the 1D pathway, implying a Mg–Mg distance of about 3 Å. Images illustrating this pathway in a supercell of zircon YVO_4_ are shown in Figure 4.

**Figure 5:**
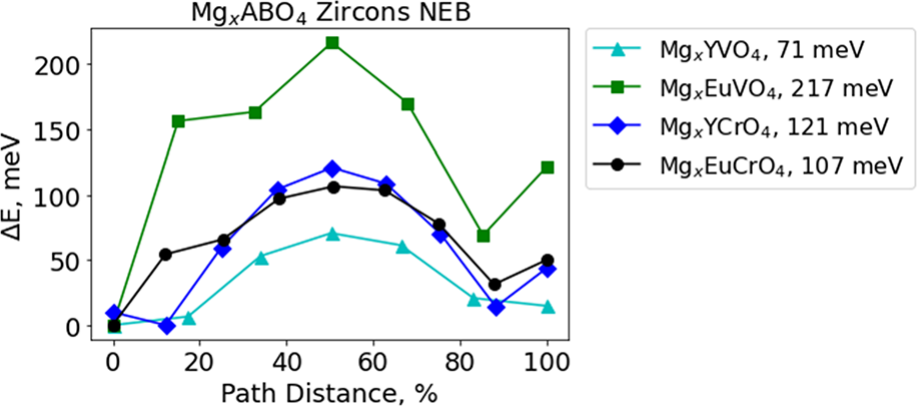
Energy profiles determined by DFT-NEB for Mg^2+^ migration across the characteristic ∼1.5 Å linear hop at the dilute lattice limit (single Mg-ion in supercell) for zircon YVO_4_, EuVO_4_, YCrO_4_, and EuCrO_4_.

DFT nudged elastic band (NEB) calculations were performed to evaluate the solid-state Mg-ion mobility along this path in the dilute limit of Mg ions, which in our supercell corresponds to one Mg per 16 formula units. The resulting change in energy as the Mg-ion traverses the linear ∼1.5 Å hop in YVO_4_, EuVO_4_, YCrO_4_, and EuCrO_4_ is shown in Figure 5. The Mg-ion dilute lattice limit migration barrier is 71 meV for YVO_4_, 217 meV for EuVO_4_, 121 meV for YCrO_4_, and 107 meV for EuCrO_4_. These migration barrier values are all remarkably low for Mg^2+^. As a point of comparison, previous work has reported that migration barriers <650 meV would correspond to intrinsic ionic mobility sufficient for room temperature C/2 cycling with nanosized particles.^8^ The dilute Mg-ion migration barrier was calculated using the same methods for Chevrel Mg_*x*_Mo_6_S_8_, and the first prototype Mg cathode is 565 meV. Chevrel Mg_*x*_Mo_6_S_8_ is the first Mg cathode experimentally demonstrated to have sufficient rate capability for repeated cycling at room temperature.^1,33^ To our knowledge, the lowest calculated Mg^2+^ migration barrier that has ever been reported is ∼80 meV, for a theoretical, to date unrealized compound, Mo_3_(PO_4_)_3_O.^34^ It is encouraging that high Mg-ion mobility is consistently predicted across the broader zircon family and is not limited to a specific chemistry. This suggests that these transport properties are connected to the unique structural characteristics of this family of compounds.

### Experimental Synthesis of Zircons

**Figure 6:**
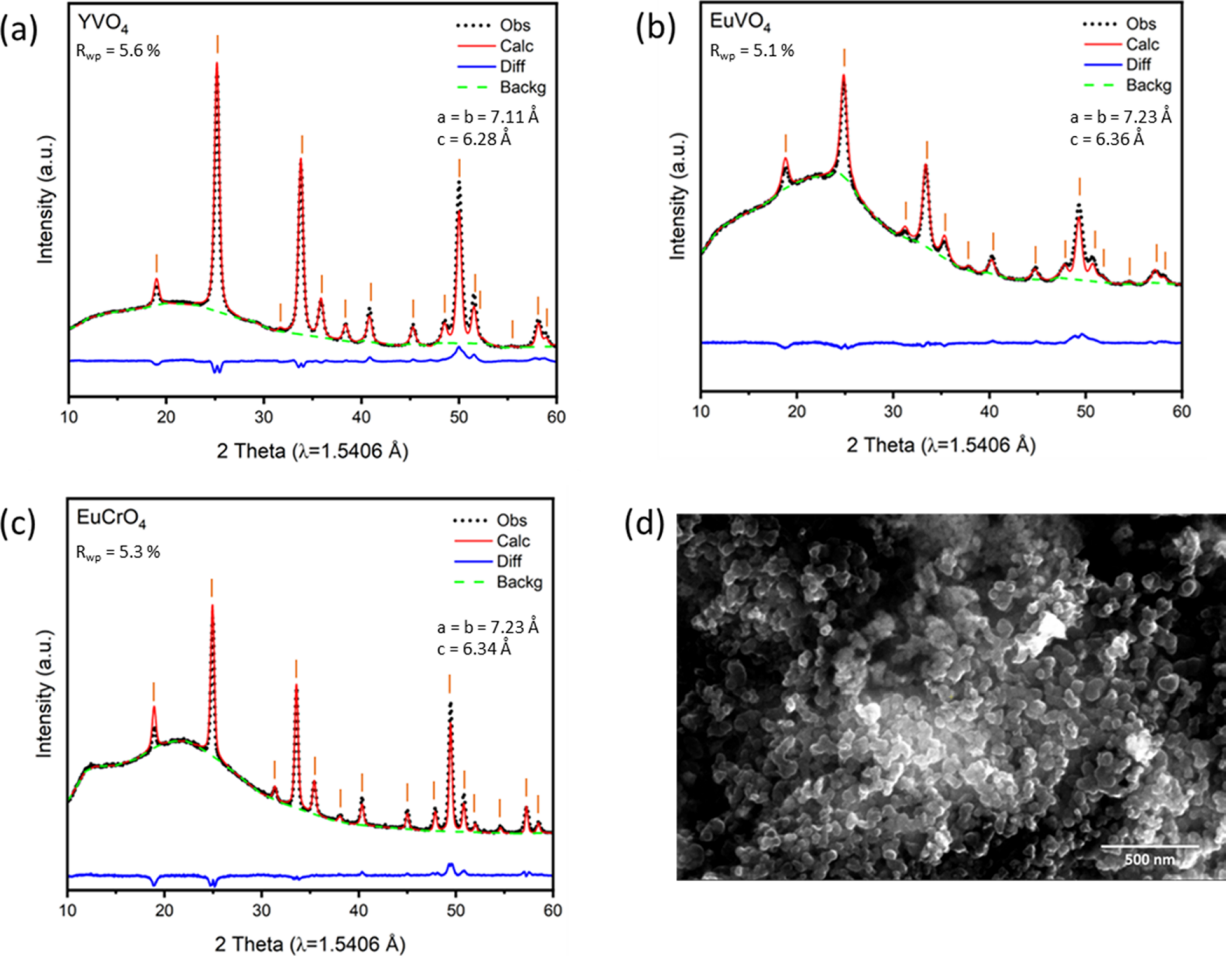
XRD results of the as-synthesized zircon (a) YVO_4_, (b) EuVO_4_, and (c) EuCrO_4_ compounds. (d) SEM image of synthesized zircon YVO_4_ sample after calcination at 500 °C for 30 min.

The sol–gel technique has been shown to be successful in the synthesis of YVO_4_ nanopowders.^35^ In this study, a similar recipe was used to synthesize three different zircon ABO_4_ compounds (YVO_4_, EuVO_4_, and EuCrO_4_) using oxide precursors as the source of the B-site transition metals (V, Cr) and nitrate-based precursors as the source of A-site 3+ cations (Y, Eu). Further synthesis details are included in the “Methods” section. The phase purity of the synthesized samples was evaluated through X-ray diffraction (XRD) studies and subsequent Rietveld refinement analysis, with the results shown in Figure 6. The XRD patterns of zircon YVO_4_, EuVO_4_, and EuCrO_4_ all indicate high phase purity with no detectable crystalline impurities. The synthesis calcination time was limited to 30 min for 500 °C to obtain smaller particle sizes with a homogeneous distribution compared to classic solid-state synthesis techniques (which require long sintering times of 20–30 h at high temperatures such as 800 °C). A sample scanning electron microscopy (SEM) image of YVO_4_ is provided in Figure 6d. SEM images of the zircon YVO_4_, EuVO_4_, and EuCrO_4_ samples revealed that all samples had a homogeneous particle size distribution with an average particle size range of 50–60 nm.

### Electrochemical Cycling of Synthesized Zircons as Mg Cathodes

**Figure 7:**
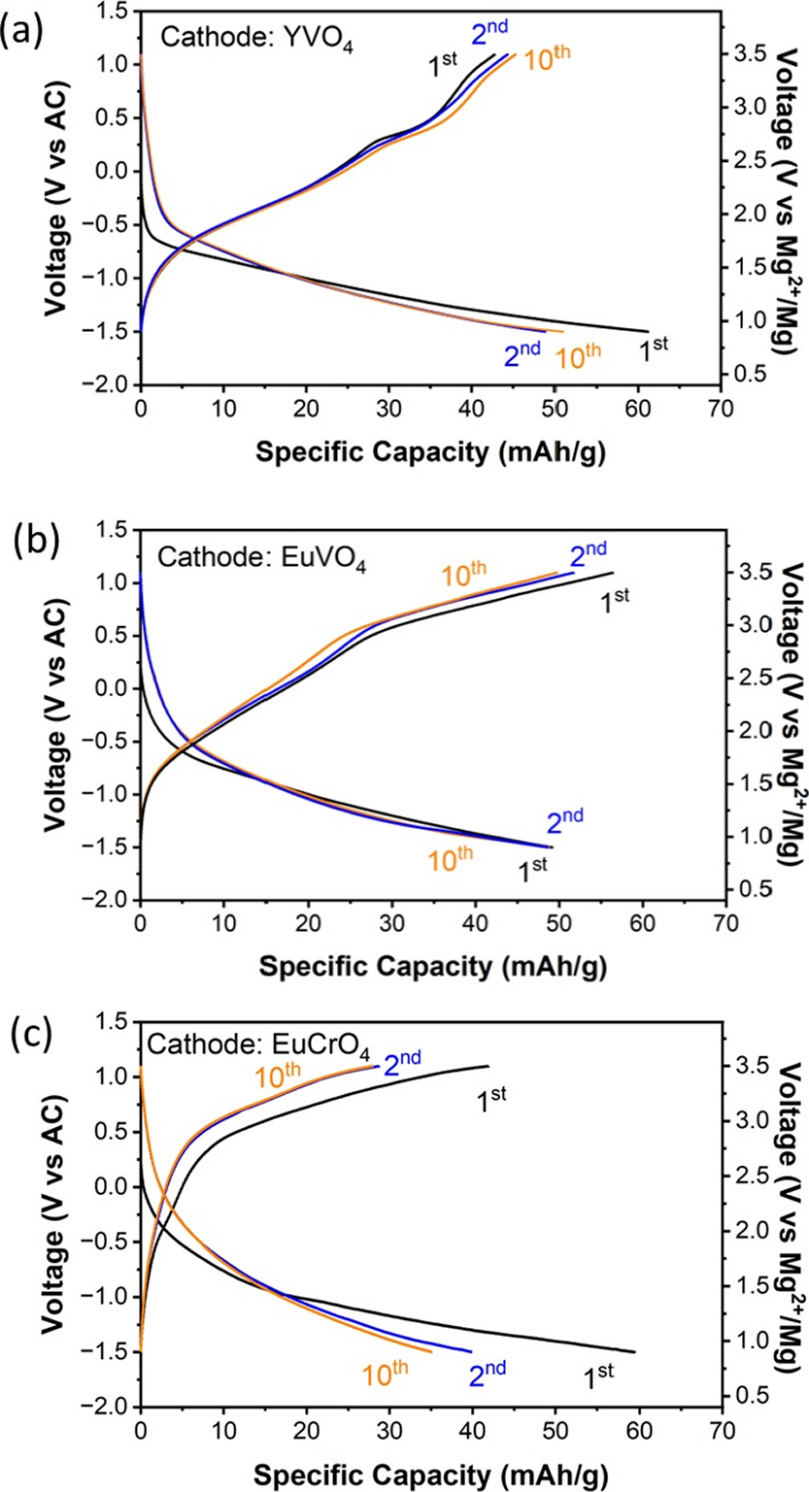
Experimentally measured charge and discharge voltage profiles of zircon (a) YVO_4_, (b) EuVO_4_, and (c) EuCrO_4_ as Mg cathodes against activated carbon anodes at 50 °C with a current density of 2 mA/g.

Coin cells were prepared for the synthesized zircon YVO_4_, EuVO_4_, and EuCrO_4_ samples. The coin cells used an activated carbon (AC) anode and custom electrolyte, 0.5 M Mg(TFSI)2 in 1 M diglyme in 1,1,2,2-tetrafluoroethyl-2,2,3,3-tetrafluoropropyl ether (TTE). The coin cells were tested at 50 °C with a current density of 2 mA/g in the voltage range of −1.5 to 1.1 V vs AC. The resulting electrochemical cycling data for all three samples under the same cycling protocol and conditions are shown in Figure 7. The first cycle measured discharge capacities were 62 mAh/g for YVO_4_, 50 mAh/g for EuVO_4_, and 59 mAh/g for EuCrO_4_. The 2nd cycle measured discharge capacities were 43 mAh/g for YVO_4_, 55 mAh/g for EuVO_4_, and 42 mAh/g for EuCrO_4_. The 10th cycle measured discharge capacities were 50 mAh/g for YVO_4_, 48 mAh/g for EuVO_4_, and 30 mAh/g for EuCrO_4_. YVO_4_ and EuCrO_4_ showed notable capacity losses after the first cycle while EuVO_4_ showed the best capacity retention

Generally, the vanadium-based zircons showed better reversible cycling compared to EuCrO_4_, which exhibited the lowest capacity retention of the three samples. XRD performed on EuCrO_4_ and YVO_4_ after electrochemical cycling confirmed that the tetragonal zircon phase was the only crystalline phase present after cycling. These results are included in the Supporting Information. The ex situ XRD verified that the poor performance of EuCrO_4_ is not related to any significant bulk phase transition upon cycling, though it cannot exclude whether some part of the material dissolves in the electrolyte or makes an amorphous product.

### Confirming Mg Intercalation with EDS

Ex situ SEM-EDS analysis was performed to verify Mg intercalation after electrochemical cycling in the synthesized zircon YVO_4_, EuVO_4_, and EuCrO_4_ samples. New coin cells were prepared to harvest the cathode materials for analysis after the 1st charge cycle and after the 1st discharge cycle. These ex situ SEM-EDS results are reported in Table 4. They confirm that after the 1st discharge down to −1.5 V vs AC, Mg is present in all three samples, and the Mg atomic percentages is consistent with the capacities measured from electrochemical testing. The EDS data furthermore shows that after the 1st full cycle (1st discharge then 1st charge) Mg is successfully extracted in all three samples (with atomic percentage < 1% remaining). Overall, the ex situ SEM-EDS measurements confirmed successful Mg intercalation in the zircon YVO_4_, EuVO_4_, and EuCrO_4_ samples.

**Table 4:** Elemental Compositions of the Ex Situ ABO_4_ Zircon Samples Collected after the 1st Discharged Cycle and the 1st Full Cycle (1st Discharge Then 1st Charge) Measured by SEM-EDS Analysis

ABO_4_ Zircon	YVO_4_		EuVO_4_		EuCrO_4_	
	After 1st Discharge	After 1st Charge	After 1st Discharge	After 1st Charge	After 1st Discharge	After 1st Charge
	Atomic Percentages (%)					
Mg	8.94	0.92	7.52	0.54	6.24	0.86
A site (Y, Eu)	44.86	48.22	45.58	49.70	47.20	50.23
B site (V, Cr)	46.20	50.86	46.90	49.76	46.56	48.91

## Discussion

**Figure 8:**
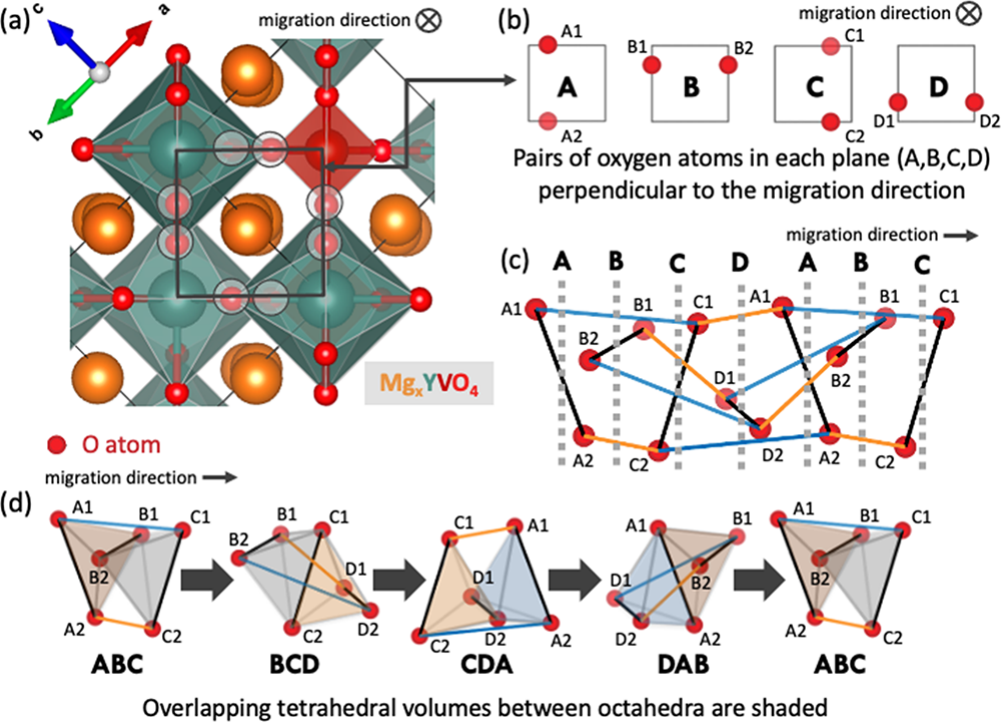
Depiction of the unique zircon structural motif, which has one-dimensional linear channels of interstitial sites composed of distorted octahedra and tetrahedrons that are overlapping in volume. The YVO_4_ zircon supercell crystal structure with the migration direction perpendicular to the plane of the page is shown in panel (a). The same perspective is used in (b) to depict the repeating pattern of oxygens along the channel, which can be divided into pairs of atoms (A, B, C, and D), which rotate along the migration direction. This repeating pattern of oxygen atoms where the migration direction is within the plane of the page is shown in (c) with the addition of solid colored (orange, blue, and black) lines to represent distances of the same length and dashed gray lines marking the pairs of oxygen atoms (A, B, C, and D). The distorted octahedra sites along the diffusion pathway are shown in (d) with shading to show the overlapping volumes from four shared atoms (forming a tetrahedra) between distorted octahedra.

We propose that the remarkably high Mg-ion mobility predicted based on these reported NEB results is due to the unique structural motif found in ABO_4_ zircon materials. As previously introduced, the zircon structure exhibits one-dimensional channels of interstitial sites composed of overlapping, distorted octahedra and tetrahedrons.^12^ This one-dimensional channel in the zircon structure (see Figure 8) enables ionic transport via unusually short, repeating ∼1.5 Å hops between interlocking octahedra and tetrahedra. Oxygens form a repeating pattern along the channel and can be divided into pairs of atoms (A, B, C, and D), which rotate as one moves along the migration direction which is depicted in Figure 8b,c. The interstitial distorted octahedral sites can be visualized by considering three adjacent pairs of oxygens (six atoms total), while the tetrahedral sites are formed by two adjacent pairs of oxygens (four atoms total). Figure 8d shows the two tetrahedral sites (e.g., AB and BC) contained within one distorted octahedra (e.g., ABC) which are shaded. Two neighboring distorted octahedral sites (e.g., ABC and BCD) share four atoms (e.g., B1, B2, C1, and C2) and overlap in volume through the shared tetrahedral site (BC).

Furthermore, the distortion of the octahedral interstitial site in the zircon structure reduces the preference of the Mg-ion for this site. Minimizing the change in coordination of the migrating ion along the diffusion pathway correlates with smaller site energy differences, resulting in favorable, lower migration barriers because of the resulting flatter energetic landscape.^8,34^ Large changes along the path to lower coordination numbers, such as 2 and 3, have been shown to correspond to the most unfavorable sites along a diffusion pathway for multivalent ions in a variety of materials.^2,8,34^ This makes overlapping distorted octahedral and tetrahedral interstitial sites of zircon particularly well suited for Mg-ion transport. The interlocked interstitial sites of the one-dimensional zircon diffusion channels result in a “6-5-4” change in coordination, which corresponds to significantly less coordination change as compared to the typical “6-3-4” change in coordination found in diffusion pathways composed of face-sharing tetrahedral and octahedral sites (see Figure 1). The intermediate coordination of 5 in the zircon structure is much more favorable than 3 because migrating ions avoid squeezing through a plane of anions, which usually corresponds to higher energies.^2^

While the Mg-ion transport properties of the zircon family are attractive, this structure presents other disadvantages when considered as an intercalation cathode. The zircon structure exhibits one-dimensional diffusion channels which usually requires nanosized primary particles in practical applications, due to the inevitable blocking of the transport passages by intrinsic anti-site defects in larger particles.^36^ Indeed, our first attempts to synthesize the zircon materials by solid-state methods resulted in micron-sized particles, which exhibited very poor electrochemical performance. Furthermore, the theoretically predicted and experimentally measured voltages are also too low to be attractive as high-performance Mg cathodes. Finally, in addition to these limitations, DFT calculations predicted poor phase stability for zircon materials upon Mg-ion intercalation, except for EuVO_4_.

We suggest that the poor phase stability of zircon materials when discharge is connected to the reduction of the tetrahedral transition metal (B atom) in the ABO_4_ zircon structure. Small, higher valence transition metals (e.g., V^5+^, Cr^6+^, and Cr^5+^) favor tetrahedral coordination while lower valence transition metals (e.g., V^4+^, V^3+^, Cr^4+^, and Cr^3+^) favor octahedral coordination.^7^ Therefore, when the redox active transition metal is reduced to a lower oxidation state upon Mg-ion intercalation (V^5+^ → V^4+^ or Cr^5+^ → Cr^4+^), the tetrahedral coordination becomes less stable and cation migration into the diffusion channel is likely.

While zircon EuVO_4_ demonstrated the most stable electrochemical performance and capacity retention, its experimentally measured capacity (∼50 mAh/g) is still lower than expected (100 mAh/g). Zircon YVO_4_ exhibited the highest first cycle discharge capacity (62 mAh/g) of the three synthesized zircons, however, the measured capacity is lower than expected. The higher initial capacity measured for YVO_4_ correlates with its highest theoretical capacity (263 mAh/g for Mg + YVO_4_ ⇔ MgYVO_4_) which is attributed to the significantly smaller atomic weight of Y compared to Eu and the higher maximum intercalation level predicted. This initial work focuses on evaluating the viability of zircon materials as Mg cathodes, but further investigations into the causes behind the limited electrochemical performance of these materials are clearly needed.

## Conclusions

In conclusion, this work evaluated the viability of 4 zircons (YVO_4_, EuVO_4_, YCrO_4_, EuCrO_4_) as Mg intercalation cathodes with DFT. Among these materials, three (YVO_4_, EuVO_4_, and EuCrO_4_) were successfully synthesized and experimentally tested to validate their electrochemical properties. While all four zircon compounds were calculated to exhibit remarkably good Mg-ion transport with low Mg^2+^ migration barriers (<250 meV), zircon EuVO_4_ has the best predicted phase stability and electrochemical performance upon experimental testing. The promising Mg-ion transport properties of the zircon family are attribute to their unique “6-5-4” change in coordination of the migrating ion along the diffusion pathway, which is created by overlapping interstitial distorted octahedral and tetrahedral sites. As such, the zircon structure presents exciting design motifs for promoting Mg-ion mobility; however, the one-dimensional diffusion pathways, limited voltages, and tetrahedral coordination of the redox-active transition metal likely limit their viability as suitable Mg cathodes. While zircons may not serve as promising high-performance Mg cathodes, the structure family offers useful insights into material design rules based on polyhedra with overlapping volumes for improving multivalent ion transport.

## Methods

### DFT Calculations for Phase Stability and Electrode Properties

Unit cell structures of zircon YVO_4_ (mp-19133), EuVO_4_ (mp-22796), YCrO_4_ (mp-18825), and EuCrO_4_ (mp-22586) were sourced from the Materials Project database.^14^ Single Mg atoms were inserted into each unit cell structure (corresponding to a composition of Mg_0.5_ABO_4_) before performing a DFT relaxation based on the insertion algorithm^25^ which has been implemented as a workflow in the Python package, atomate.^37^ If the host framework remained intact according to the structure matching capabilities in pymatgen,^26^ a second Mg atom was inserted into the unit cell structure. A maximum of two Mg atoms (corresponding to a composition of MgABO_4_) insertions were attempted to avoid exceeding the redox capabilities of the material where the B transition metal cannot be further reduced than B^5+^ → B^3+^. Theoretical voltages were calculated using Δ*G*_rxn_ = −*nFV*, which represents the energy difference of the intercalation reaction. Δ*G*_rxn_ is determined using the energies from DFT. *F* is Faraday’s constant and *n* = 2 for a Mg-ion intercalation reaction.

DFT relaxations were performed using the Vienna Ab initio Software Package (VASP) with the exchange correlation approximated with the Perdew–Burke–Ernzerhof (PBE) generalized gradient approximation (GGA). Hubbard *U* corrections of *U*_V_ = 3.25 and *U*_Cr_ = 3.7 eV were applied to match the Materials Project data and “MPRelaxSet” in pymatgen.^26^ “MPRelaxSet” in pymatgen^26^ was used to set the pseudopotentials used for the DFT relaxations. The total energy was sampled using a Monkhorst–Pack mesh with *k*-point density of 64 Å^–3^. Projector augmented-wave theory combined with a well-converged plane-wave cutoff of 520 eV was used to describe the wave functions. The convergence threshold of the total energy was set to 0.00005 eV/atom and a force tolerance of 0.05 eV/Å.

### NEB + DFT Calculations

NEB + DFT was used for calculating the migration barriers of zircon YVO_4_, EuVO_4_, YCrO_4_, and EuCrO_4_. These calculations were performed using VASP with the addition of Transition State Tools for VASP software.^38^ 2 × 2 × 2 supercells were created from the unit cell structures and then transformed to orient the linear diffusion pathway and one-dimensional channels in the zircon structure along the *b*-axis. The migration barrier was evaluated at the dilute lattice limit where there is a single Mg atom in the host framework, which resulted in supercell structures with a total of 97 atoms. The supercell lattice parameters were all >10 Å to eliminate the possibility of any fictious self-interaction effects on the migration ion due to periodic boundary conditions.

“MPRelaxSet” in pymatgen^26^ was used to set the DFT calculation parameters with the following exceptions. No Hubbard *U* corrections were applied as there is no conclusive evidence that GGA+*U* performs better than GGA when investigating ion migration with NEB.^39−42^ Gaussian smearing was used. No symmetry but Ψ_**k**_ = Ψ*_–k_ was assumed to reduce sampling of the Brillouin zone. An additional support grid for the evaluation of the augmentation charge was applied. A minimum of four electronic self-consistency steps were required. Endpoint structure relaxations were converged with a total energy of 0.00005 eV and a force tolerance of 0.01 eV/Å cut-off criteria. A linear interpolation of images was used between the relaxed endpoints. During the NEB calculation, images were converged to a total energy of 0.00005 eV and a force tolerance of 0.05 eV/Å cut-off criteria.

### Sol–Gel Synthesis

The synthesis method was derived from previous work on YVO_4_ nanopowders.^35^ Zircon EuCrO_4_, YVO_4_, and EuVO_4_ compounds were synthesized via sol–gel technique using stoichiometric ratios of Eu:Cr, Y:V, and Eu:V precursors, respectively. Europium nitrate pentahydrate (Eu(NO_3_)_3_·5H_2_O), yttrium nitrate hexahydrate (Y(NO_3_)_3_·6H_2_O), vanadium pentoxide (V_2_O_5_), and chromium(VI) oxide (CrO_3_) powders were purchased from Sigma-Aldrich and used without further purification. For gelation, the powders of the transition metal source depending on the targeted compound were slowly dissolved in hydrogen peroxide (H_2_O_2_ from Sigma-Aldrich). For the synthesis of EuVO_4_ and YVO_4_, 0.3 g of V_2_O_5_ was placed in a glass beaker, and 25 mL H_2_O_2_ was added dropwise. The addition of H_2_O_2_ was done very slowly to prevent excessive bubbling and loss of the material. After 10 min, a red solution was formed, and then nitrate-based Y(NO_3_)_3_·6H_2_O (for the synthesis of YVO_4_) or Eu(NO_3_)_3_·5H_2_O (for the synthesis of EuVO_4_) was added. The amount of nitrate precursor was calculated according to the targeted 1:1 ratio of Y:V or Eu:V, respectively. For this purpose, 1.263 g of Y(NO_3_)_3_·6H_2_O and 5 g of citric acid were added to the red solution. The resultant mixture was placed in a hot-plate and continuously stirred at 60 °C until it formed a viscous blue-colored gel. As a final step, the gel was collected and placed in an alumina crucible and calcined at 500 °C for 30 min. The same process was applied to synthesize zircon EuCrO_4_ by changing the precursors accordingly.

### XRD and SEM-EDS Characterization

The phase identification of the synthesized zircon EuCrO_4_, YVO_4_, and EuVO_4_ samples, and the structural changes upon cycling as Mg cathodes were observed by ex-situ XRD using a Rigaku MiniFlex 600 diffractometer with Cu K_α_ radiation (λ = 1.54178 Å) in the 2θ range of 10°–60°. Rietveld refinement was performed using the PANalytical X’pert HighScore Plus software. EDS analysis and SEM images were collected using a Zeiss Gemini Ultra-55 analytical field-emission SEM at the Molecular Foundry at Lawrence Berkeley National Lab.

### Electrochemical Cycling

Cathode films were prepared by mixing a 7:2:1 ratio of the zircon active material, carbon black (Timcal, SUPER C65), and polytetrafluoroethylene (PTFE from DuPont, Teflon 8A). Anode films were prepared by mixing a weight ratio of 8:1:1 of activated carbon (Sigma), carbon black (Timcal, SUPER C65), and PTFE (DuPont, Teflon 8A). The mixtures were rolled to form thin film electrodes with surface areas of 1 cm^2^. Coin cells were prepared with a loading density of 3 mg/cm^2^ for the cathode and 20 mg/cm^2^ for the anode. All work was performed in an argon-filled glovebox.

The electrolyte was prepared by drying magnesium(II) bis(trifluoromethanesulfonyl)imide (Mg(TFSI)_2_ from Solvionic with 99.5% purity) salt at 170 °C overnight in an argon-filled glovebox. The dried salt was then used to form a 0.5 M Mg(TFSI)_2_, 1 M diglyme (99.5%, Sigma-Aldrich) solution in 1,1,2,2-tetrafluoroethyl-2,2,3,3-tetrafluoropropyl ether (TTE from TCI Chemicals). The electrolyte and its components were always kept in an argon-filled glovebox.

Electrochemical testing was performed in coin cells made from the cathode and anode thin films using a Whatman glass microfiber filter along with the prepared 0.5 M Mg(TFSI)_2_ and 1 M diglyme in TTE electrolyte. Galvanostatic cycling was performed at 50 °C using an Arbin battery tester. The coin cells were cycled with a current density of 2 mA/g. Ex situ samples were collected after disassembling the coin cells and washing the cathode thin films with diglyme in an argon-filled glovebox.

## Supporting Information Available

SEM-EDS data on zircon EuCrO_4_ sample after electrochemical cycling; ex situ XRD data on zircon EuCrO_4_ sample after electrochemical cycling; and ex situ XRD data on zircon YVO_4_ sample after electrochemical cycling (PDF)
