# Supplementary material for: Novel Structural Motif To Promote Mg-Ion Mobility: Investigating ABO4 Zircons as Magnesium Intercalation Cathodes
Source: ACS Appl Mater Interfaces. 2023 Jul 11;15(29):34983–91. doi: 10.1021/acsami.3c05964 (PMC10375429; doi:10.1021/acsami.3c05964)
Supplement: Supplementary file 1 — am3c05964_si_001.pdf [file am3c05964_si_001.pdf]

Supporting Information

A Novel Structural Motif to Promote Mg-ion Mobility: Investigating ABO_4 Zircons as Magnesium Intercalation Cathodes

Ann Rutt[a], Dogancan Sari[a], Qian Chen[b], Jiyoon Kim[a], Gerbrand Ceder[a,b], Kristin A. Persson[a,b*]

[a] Department of Materials Science and Engineering, University of California, Berkeley, USA
[b] Materials Sciences Division, Lawrence Berkeley National Laboratory, Berkeley, USA
[*]Corresponding author can be contacted at kapersson@lbl.gov

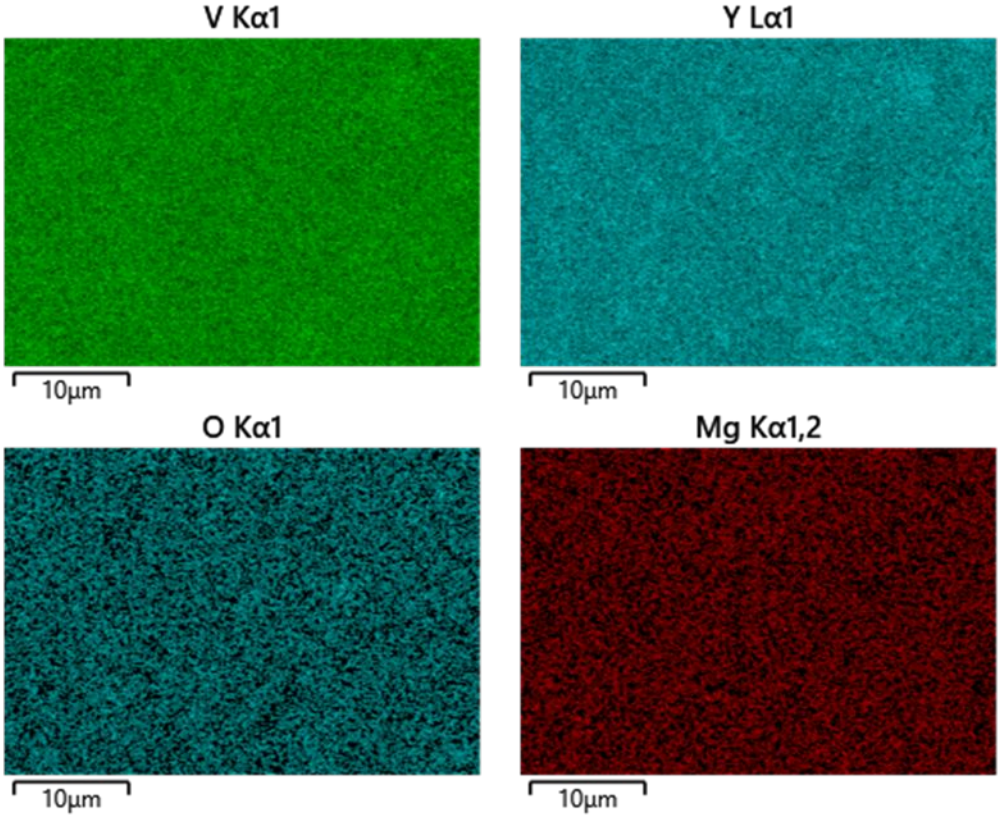

Element	Line Type	Apparent Concentration	k Ratio	Wt%	Wt% Sigma	Atomic %
Mg	K series	0.47	0.00315	3.31	0.06	8.94
V	K series	9.26	0.09264	35.88	0.11	46.20
Y	L series	15.34	0.15338	60.80	0.12	44.86

Figure S1. SEM-EDS composition mapping images and data on a sample of zircon $EuCrO_4$ harvested after electrochemical cycling.

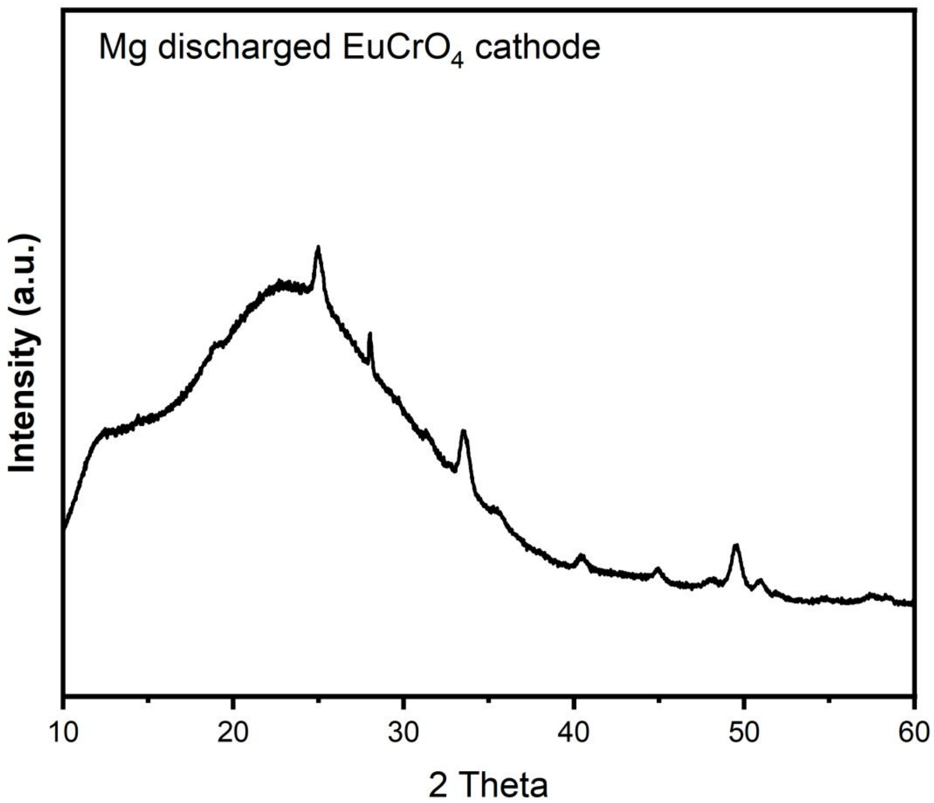

Figure S2. Ex-situ XRD data on a sample of zircon $EuCrO_4$ harvested after electrochemical cycling.

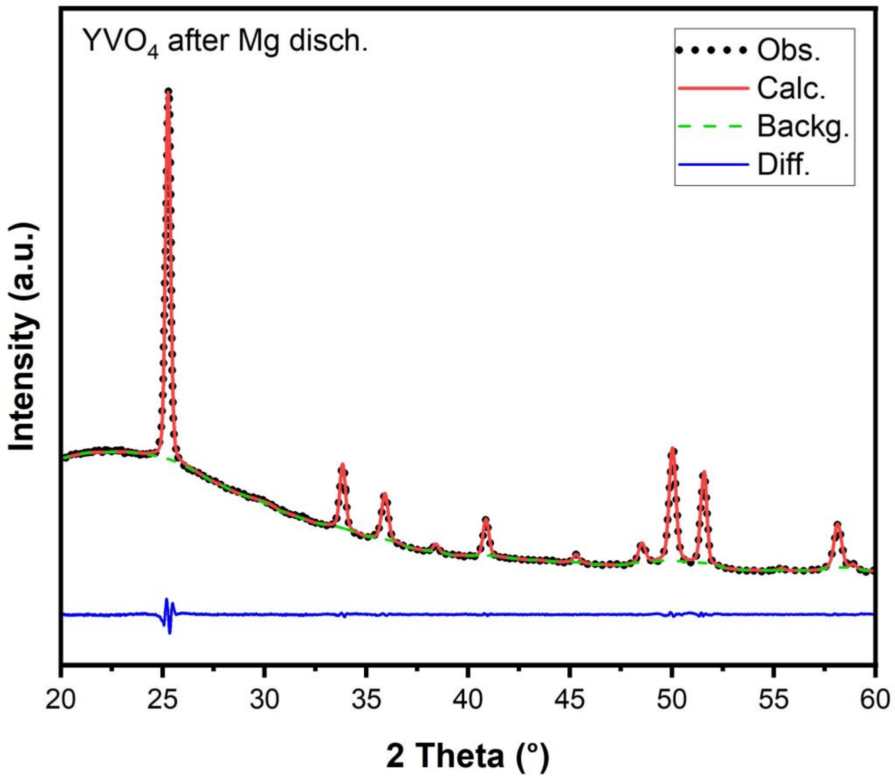

Figure S3. Ex-situ XRD data and corresponding Rietveld refinement on a sample of zircon YVO_4 harvested after the initial Mg insertion (fully discharged).
